# Cholinergic-mediated coordination of rhythmic sympathetic and motor activities in the newborn rat spinal cord

**DOI:** 10.1371/journal.pbio.2005460

**Published:** 2018-07-09

**Authors:** Mélissa Sourioux, Sandrine S. Bertrand, Jean-René Cazalets

**Affiliations:** Université de Bordeaux, CNRS UMR 5287, Bordeaux, France; Institut de Biologie Paris-Seine, France

## Abstract

Here, we investigated intrinsic spinal cord mechanisms underlying the physiological requirement for autonomic and somatic motor system coupling. Using an in vitro spinal cord preparation from newborn rat, we demonstrate that the specific activation of muscarinic cholinergic receptors (mAchRs) (with oxotremorine) triggers a slow burst rhythm in thoracic spinal segments, thereby revealing a rhythmogenic capability in this cord region. Whereas axial motoneurons (MNs) were rhythmically activated during both locomotor activity and oxotremorine-induced bursting, intermediolateral sympathetic preganglionic neurons (IML SPNs) exhibited rhythmicity solely in the presence of oxotremorine. This somato-sympathetic synaptic drive shared by MNs and IML SPNs could both merge with and modulate the locomotor synaptic drive produced by the lumbar motor networks. This study thus sheds new light on the coupling between somatic and sympathetic systems and suggests that an intraspinal network that may be conditionally activated under propriospinal cholinergic control constitutes at least part of the synchronizing mechanism.

## Author summary

Physical movements require mobilization of animals’ autonomic nervous system, in order to maintain stable bodily functions while matching the increasing physiological demand. These autonomic responses rely on a coupling between the sympathetic and somatic nervous systems, although how this coupling occurs remains unresolved. To address this issue, we used the in vitro isolated spinal cord preparation from newborn rats, which can be kept alive up to 12 h and generate locomotion-like activity. We found that the stimulation of the muscarinic cholinergic receptors specifically activates intraspinal neural networks generating a slow motor rhythm. During this slow rhythm, recordings from sympathetic neurons (the cells responsible for the spinal sympathetic output) and somatic motoneurons (responsible for skeletal muscle activity) reveal that both cell types receive a common synaptic input that results in a coupling of sympathetic and rhythmic locomotor activities. This shared somato-sympathetic drive could merge with and modulate the locomotor synaptic drive produced by the lumbar motor networks. We propose that the coupling mechanism we describe here underlies part of the vascular changes needed to maintain adequate muscle oxygenation during locomotion.

## Introduction

Locomotion, or any other form of physical activity, mobilizes not only the motor nervous system but also the autonomic nervous system, which governs body homeostatic processes such as blood pressure control and respiratory frequency. These autonomic responses that confront metabolic expenditure during exercise rely mostly on a functional coupling between the sympathetic and somatic motor systems. However, the neural substrate for this coupling remains enigmatic. Although the crucial role of supraspinal structures in this adaptive process, such as the rostral ventrolateral medulla, the hypothalamus, and the nucleus of the solitary tract, has been established [1,2], the involvement of intrinsic spinal mechanisms, albeit acknowledged, remains unclear (for review, see [3]). Significantly, several studies have reported that an intraspinal coordination between sympathetic and motor outflow may still occur in newborn [4] and adult rats [5] and mice [6] after removal of supraspinal influences by spinal cord sectioning.

The cells responsible for the spinal sympathetic outflow are the sympathetic preganglionic neurons (SPNs), which in turn innervate postganglionic sympathetic neurons. Studies on SPN membrane properties have revealed that these cells can express spontaneous rhythmic burst activity at frequencies ranging from 0.1 to 10 Hz. Each of these frequencies has been related to a specific physiological rhythm such as the cardiac and respiratory rhythms or low frequency changes in blood pressure [7–9]. Intracellular recordings from in vitro preparations or anesthetized animals have also revealed that different SPN rhythmic activity patterns can be elicited under various conditions, relying mainly on neuromodulatory controlling influences [8,10–17]. Similarly, spinal motoneurons (MNs) also fire rhythmic bursts of action potentials when engaged in locomotor activity and, as for SPNs, this rhythmogenicity is initiated and regulated mainly by neuromodulatory pathways [18,19].

Other than extrinsic neuromodulatory influences originating from supraspinal centers, an intrinsic spinal neuromodulatory influence involving acetylcholine (Ach) has been shown to play an important role in controlling both the spinal motor network and sympathetic neuron activity [20–28]. In addition to SPNs and MNs that use Ach as their neurotransmitter, thoracic segments are enriched in cholinergic interneurons and show a dense expression of muscarinic cholinergic receptors (mAchRs) [29,30]. The question then arises as to the precise role of these Ach-releasing interneurons in the thoracic spinal cord region.

In the present study, by using the in vitro isolated spinal cord preparation of newborn rats, we show that an activation of mAchRs unmasks rhythmogenic capabilities of the spinal thoracic segments and a common synaptic drive to SPNs and axial MNs. This thoracic mAchR-induced rhythmicity merges with the locomotor activity expressed in lumbar spinal networks and modulates the latter's expression. We therefore propose that thoracic networks sensitive to mAchR-mediated cholinergic influences may act as an important intrinsic substrate for the coupling between spinal motor and sympathetic activities.

## Results

### Muscarinic activation of thoracolumbar networks

**Fig 1:**
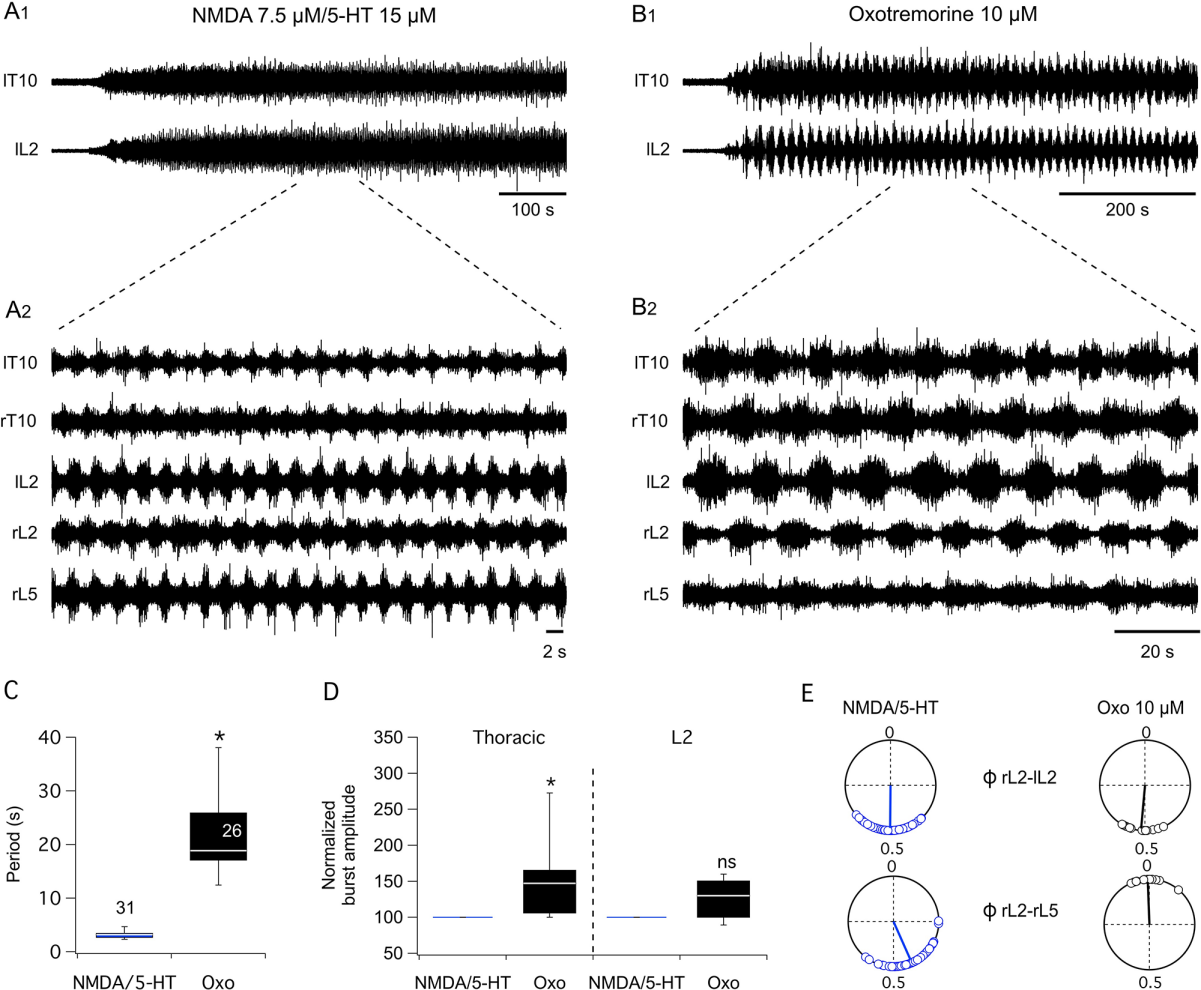
Oxotremorine-induced rhythmic bursting in the isolated newborn rat spinal cord. (A) Representative extracellular recordings from different right (r) and left (l) thoracic (T) and lumbar (L) ventral roots in the presence of NMDA/5-HT on the whole thoracolumbar spinal cord at slow (A1) and faster timescales (A2). (B) Equivalent recording setup in the same preparation with 10 μM oxotremorine bath-applied to the whole thoracolumbar spinal cord. (C) Box plots of the mean cycle period under NMDA/5-HT (blue boxes) or oxotremorine (Oxo, black boxes). (D) Box plots of normalized burst activity recorded in the presence of NMDA/5-HT (blue boxes) or oxotremorine (Oxo, black boxes). Motor burst amplitudes were normalized to the mean NMDA/5-HT burst amplitude computed in each experiment. (E) Polar graphs of rL2/lL2 and rL2/rL5 burst phase relationships under NMDA/5-HT (blue circles) or 10 μM Oxo (open circles) generated from the experiments presented in A and B. 0, in phase; 0.5, antiphase. The direction and length of vectors (blue line) indicate phase means and dispersions, respectively. *p < 0.05. Numbers in boxes indicate numbers of preparations tested. l, left; L, lumbar; NMDA, N-methyl-D-aspartate; ns, nonsignificantly different; Oxo, oxotremorine; r, right; T, thoracic; 5-HT, serotonin.

**Fig 2:**
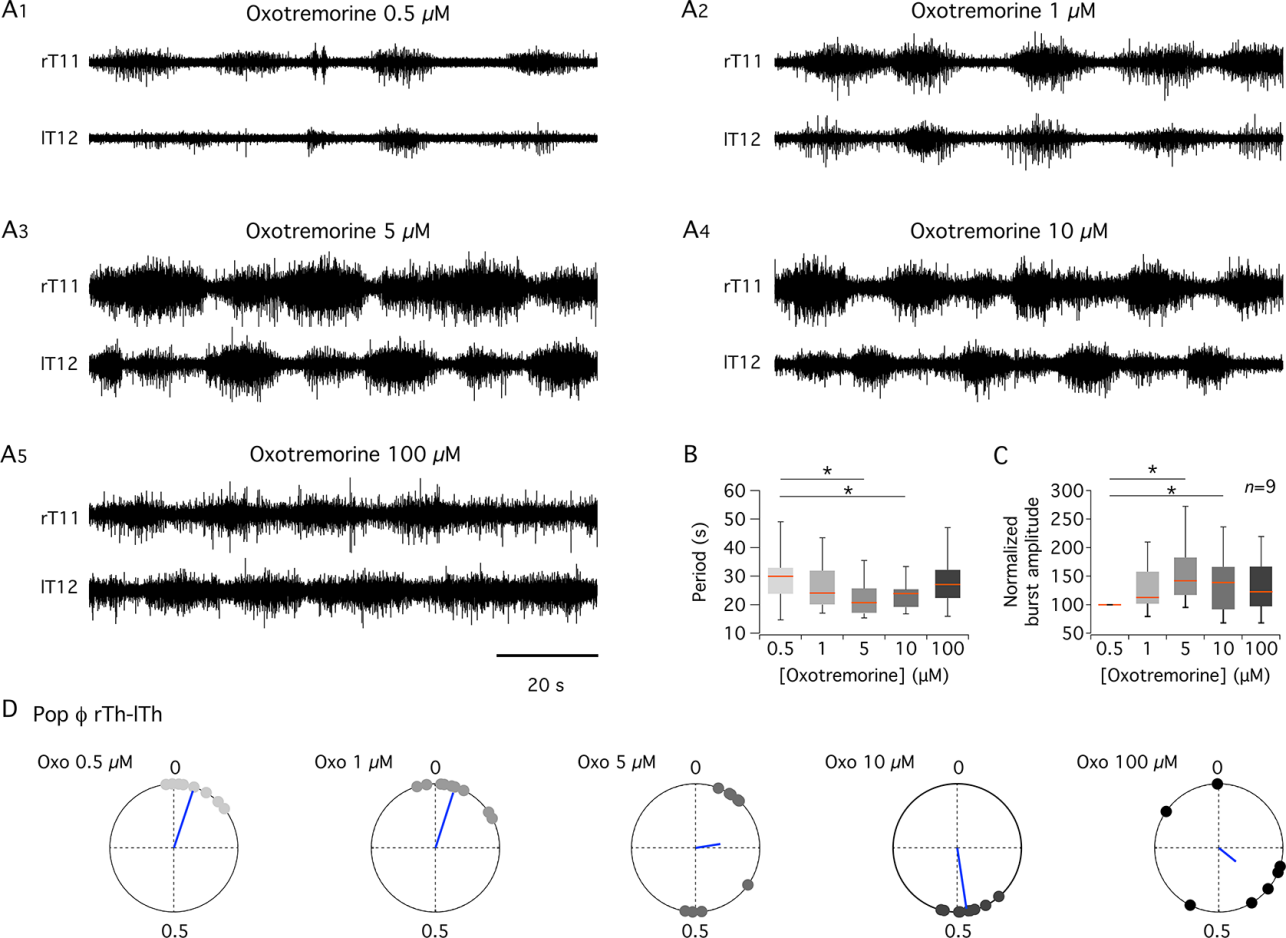
Dose-dependent effects of oxotremorine. (A) Recordings from right thoracic 11 and left thoracic 12 (rT11, lT12) ventral roots in the presence of increasing concentrations of oxotremorine bath-applied to the same whole thoracolumbar cord preparation (A1–A5). (B, C) Box plots of burst cycle period (B) and normalized amplitude (C) at different oxotremorine concentrations. Motor burst amplitudes were normalized to the mean burst amplitude computed in the presence of 0.5 μM oxotremorine in each experiment. (D) Polar graphs of the right and left alternation of thoracic motor bursts (Pop ϕ rTh-lTh) computed under increasing concentrations of oxotremorine (Oxo). 0, in phase; 0.5, antiphase. The direction and length of vectors (blue lines) indicate phase means and dispersions, respectively. Each dot on polar graphs represents one experiment. *p < 0.05. l, left; *n*, number of preparations; Oxo, oxotremorine; Pop, population; Pop ϕ rTh-lTh, right and left alternation of thoracic motor bursts; r, right; T, thoracic.

To assess the effect of cholinergic system activation on thoracic segments, we used oxotremorine, a nonselective cholinergic muscarinic agonist. In a first series of experiments, oxotremorine was bath-applied on the whole thoracolumbar spinal cord. As previously described in such preparations, a mixture of N-methyl-D-aspartate (NMDA) and serotonin (5-HT)-induced rhythmic locomotor-related activity was recorded from both lumbar and thoracic ventral roots (Fig 1A1, 2), with a mean period of 3.2 ± 0.1 s, *n* = 31 (Fig 1C) [31,32]. This fictive locomotion was characterized by right-left and extensor-flexor alternations of bursts of action potentials monitored from the L2 and L5 ventral root bursts, respectively (Fig 1E) [33]. In contrast, the bath-application of 10 μM oxotremorine on the whole spinal cord elicited a slow rhythmic activity in the thoracic and lumbar region, with a mean period of 21.7 ± 1.3 s, *n* = 26 (Fig 1B1, 2 and Fig 1C). This oxotremorine-induced rhythm consisted of right and left slow alternating bursts of action potentials, but with no alternation between the flexor and extensor units in the majority of the preparations tested (Figs 1E and 2D). Oxotremorine also was more potent in exciting thoracic segments than the NMDA/5-HT cocktail. Indeed, the amplitude of the thoracic bursts was significantly increased in the presence of oxotremorine compared to NMDA/5-HT, while L2 burst amplitudes were not significantly increased in the same conditions (Fig 1D).

This oxotremorine-induced rhythm was consistently observed at concentrations ranging from 0.5 to 100 μM (Fig 2A1–5). Increasing the oxotremorine concentration up to 10 μM progressively decreased the cycle period of thoracic motor bursts (Fig 2B) and increased their amplitude (Fig 2C). Furthermore, there was a switch from an in-phase activity recorded from the left and right segments at the lowest concentrations (0.5 and 1 μM) to an out-of-phase activity under 10 μM (Fig 2D). Five micromolar oxotremorine appears as an intermediate concentration, with in-phase left and right motor bursts observed in 55% of the preparations tested and alternating motor bursts in the remaining ones (*n* = 9) (Fig 2D). Increasing oxotremorine up to 100 μM seemed to become less effective as the induced motor activity exhibited an increased period, compared to that with 5 and 10 μM oxotremorine (Fig 2B), and a disorganized motor burst expression (Fig 2D). The various patterns elicited when raising the oxotremorine concentration could be linked to the progressive increase in the excitation state of spinal networks and recruitment of spinal neurons [34,35].

**Fig 3:**
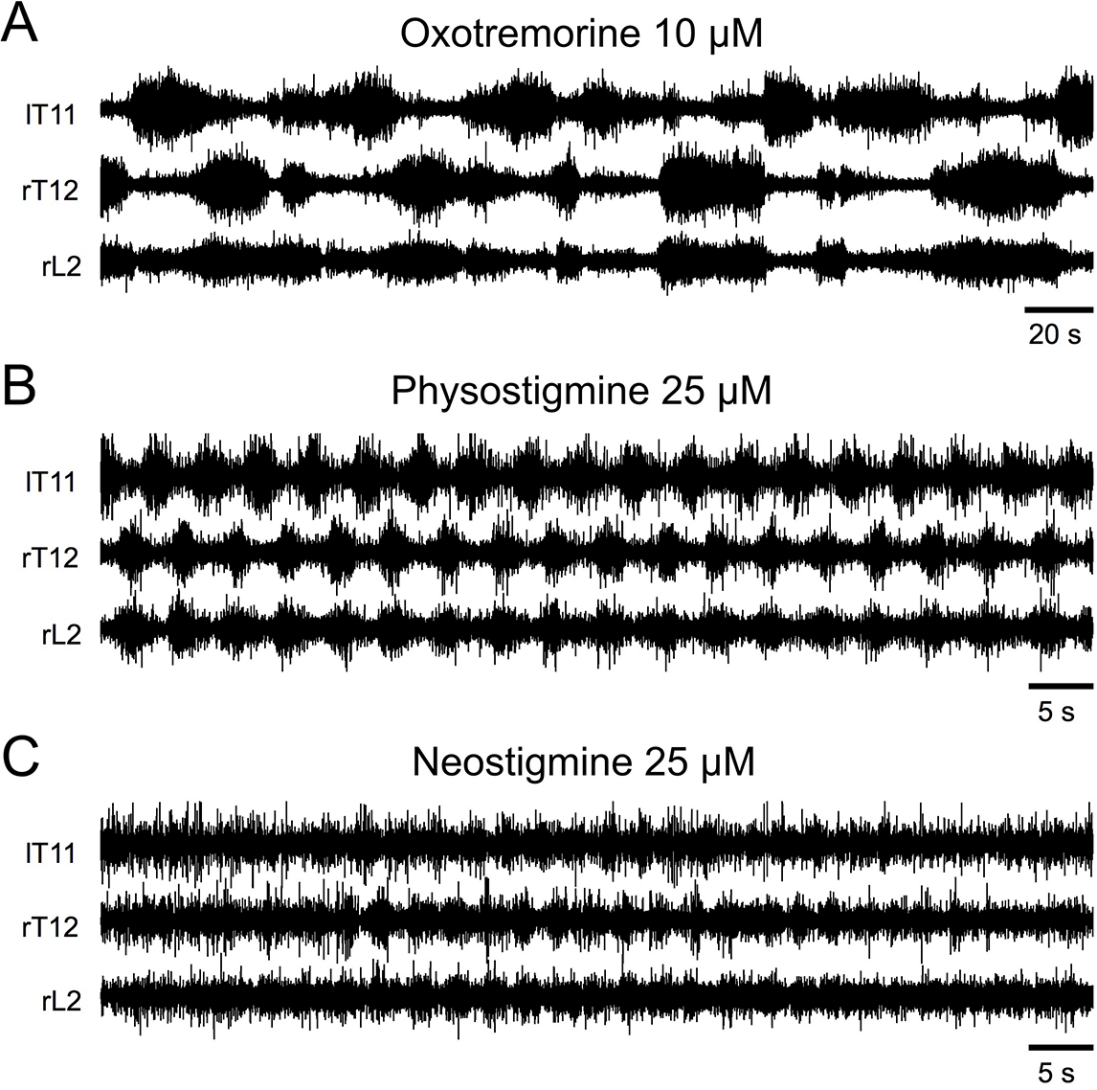
Acetylcholinesterase inhibitors elicit locomotor-like spinal cord activity. Representative extracellular recordings from left thoracic 11, right thoracic 12, and right lumbar 2 (lT11, rT12, rL2) ventral roots in the presence of oxotremorine (A) or with the acetylcholinesterase inhibitors neostigmine (B) or physostigmine (C) bath-applied on the whole thoracolumbar spinal cord. l, left; L, lumber; r, right; T, thoracic.

It has been previously shown that activation of the cholinergic system by inhibiting acetylcholinesterase (AchE), the enzyme responsible for Ach degradation with physostigmine, neostigmine, or edrophodium, elicits locomotor-like activities in lumbar ventral roots in the isolated spinal cord preparation from newborn rat [21,24,25] and that this rhythm is mediated through mAchR activation [27]. Therefore, in a next step, we compared the effects of oxotremorine (Fig 3A) with those of the AchE inhibitors neostigmine and physostigmine. In our experimental conditions, neither inhibitor mimicked the effects of oxotremorine (Fig 3B and 3C). Rather, their superfusion transiently elicited episodes of fast locomotor-like rhythmicity, as previously described [24,25,27], instead of the slow non-locomotor rhythm observed here in the presence of oxotremorine. The mean period computed in the presence of neostigmine or physostigmine was 3 ± 0.3 s (*n* = 5 preparations tested in the presence of neostigmine and *n* = 2 in the presence of physostigmine).

**Fig 4:**
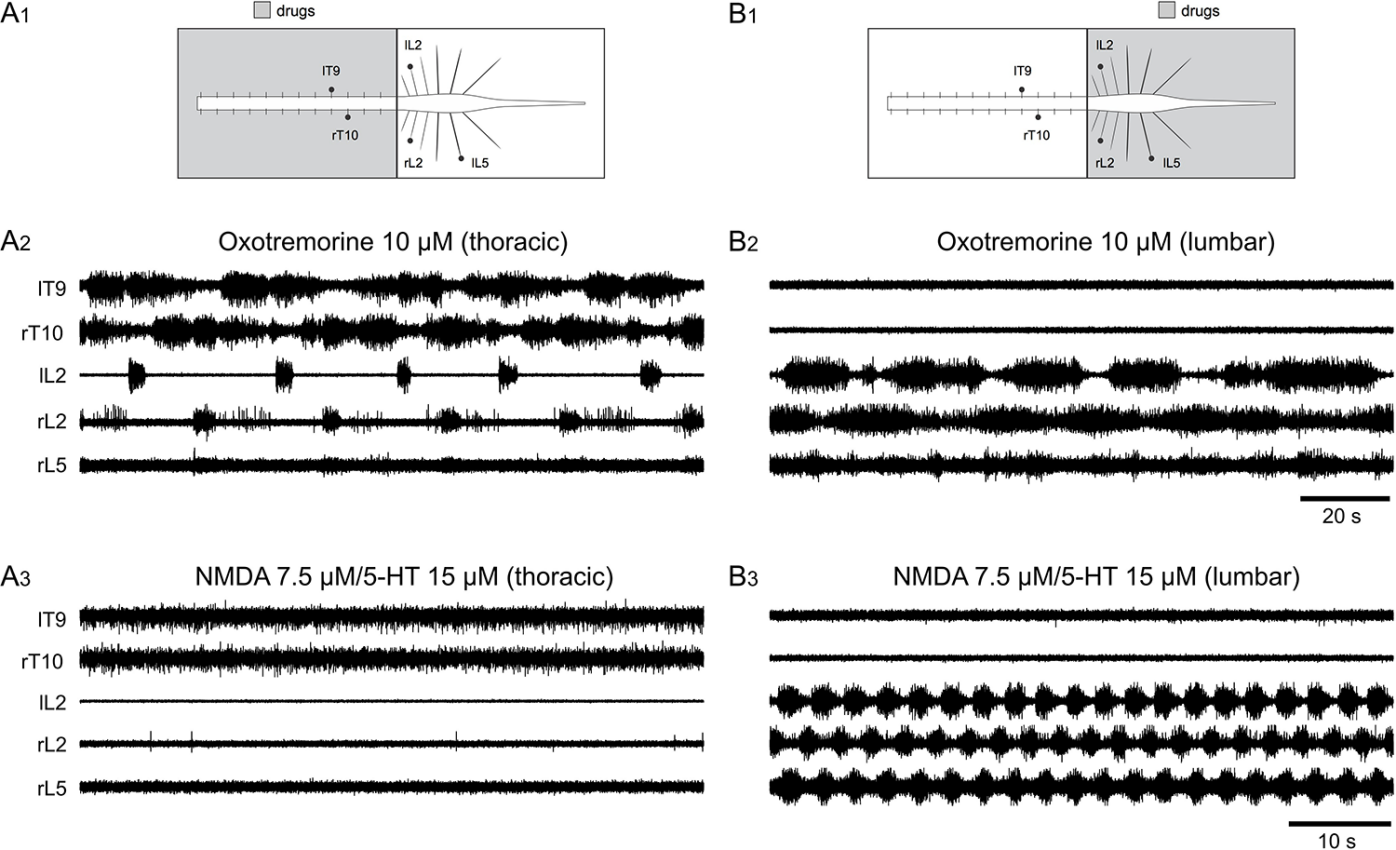
Differential sensitivity of thoracic and lumbar segments to oxotremorine and NMDA/5-HT. (A, B) Diagrams of the experimental setup for two different preparations. In both cases, a Vaseline wall (black bar) was placed at the T13 level (A1, B1). Representative recordings from left thoracic 9 (lT9), lumbar 2 (lL2), and right T10 (rT10), L2 (rL2), and L5 (rL5) ventral roots (black dots in A1 and B1) in the presence of oxotremorine in either the thoracic (A2) or lumbar compartments (B2). Same preparations in the presence of NMDA/5-HT in either the thoracic (A3) or lumbar compartments (B3). l, left; L, lumbar; NMDA, N-methyl-D-aspartate; r, right; T, thoracic; 5-HT, serotonin.

The results obtained when oxotremorine was bath-applied to the whole thoracolumbar spinal cord (Fig 1D) suggested that the thoracic and lumbar spinal levels may respond differentially to oxotremorine. To test whether the thoracic and lumbar segments exhibit similar responses to mAchR activation by oxotremorine, we used a partitioned spinal cord (see Material and methods) in which the thoracic and lumbar compartments could be independently superfused (Fig 4A1 and 4B1). Bath-application of oxotremorine to the thoracic segments alone induced a rhythmic burst pattern that was recorded from the various thoracic segments but also from lumbar roots (Fig 4A2). This thoracolumbar propagation was observed in 24 of 28 preparations tested. In contrast, and as previously shown [32], the bath-application of NMDA/5-HT to the thoracic segments alone did not induce any rhythmic activity (*n* = 10; Fig 4A3) in these experimental conditions. When the bath-application of oxotremorine was now restricted to the lumbar spinal cord, rhythmic bursting was recorded solely from the lumbar ventral roots, without any propagation to the thoracic segments (*n* = 10; Fig 4B2). The same lack of lumbo-thoracic propagation was observed when oxotremorine was replaced by NMDA/5-HT in the caudal compartment, with the recorded locomotor-like activity always restricted to the lumbar segments only (*n* = 13; Fig 4B3). It should also be noted that, using transverse sections of the thoracic spinal cord, we determined the number of thoracic segments sufficient to produce the oxotremorine-induced rhythm. In five preparations, we observed that, regardless of the thoracic level, spinal pieces of at least three thoracic segments were sufficient to generate bilateral alternating rhythmic activity in the presence of 10 μM oxotremorine. Altogether, these results suggest that the thoracic spinal cord exhibits a distinct sensitivity to oxotremorine compared to the lumbar spinal cord and that thoracic segments are endowed with specific rhythmogenic capabilities that can be unveiled by mAchR activation.

**Fig 5:**
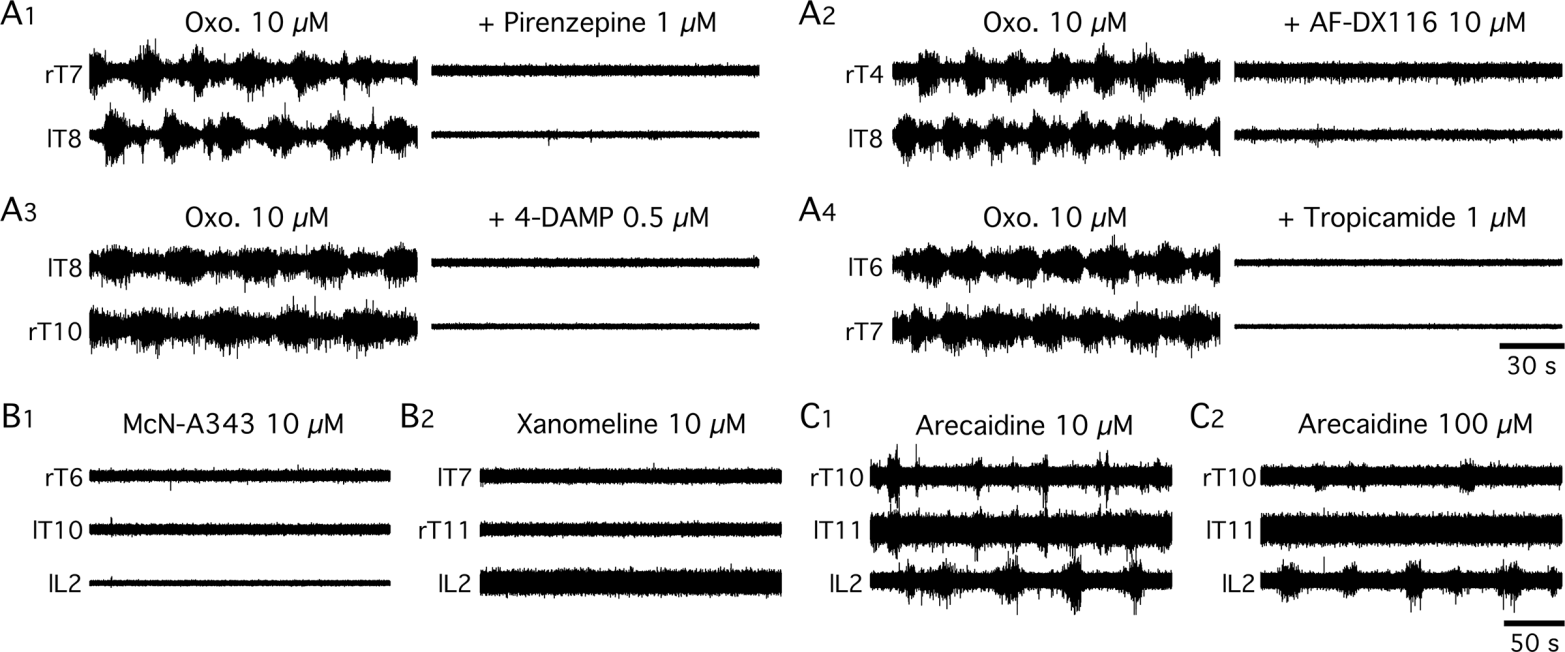
Effects of cholinergic muscarinic antagonists and agonists on the oxotremorine-induced rhythm. (A) Extracellular recordings from right (r) and left (l) thoracic (T) or lumbar (L) ventral roots in the presence of oxotremorine alone and after concomitant bath-application of oxotremorine and the M1 receptor antagonist pirenzepine (A1), or the M2 receptor antagonist AF-DX 116 (A2), or the M3 receptor antagonist 4-DAMP (A3) or the M4 antagonist tropicamide (A4). (B) The M1 agonists McN-A 343 (B1) and xanomeline (B2) failed to induce any activity. (C) Slow bursting activity elicited by bath-application of the M2 receptor agonist arecaidine at 10 μM (C1) and 100 μM (C2). l, left; L, lumbar; NMDA, N-methyl-D-aspartate; Oxo., oxotremorine; r, right; T, thoracic.

In a next step, we assessed whether and which of the four classes of mAchR subtypes present in the spinal cord [36] were involved in the genesis of the oxotremorine-induced rhythm. To this end, after a first control bath-application of oxotremorine, receptor subtype antagonists [37] were preincubated on the in vitro spinal cord preparation before their co-application with oxotremorine. An example of the blockade of the oxotremorine-induced rhythm by the presence of the antagonist of the M1 receptor subtype (pirenzepine 1–10 μM, *n* = 6) is provided in Fig 5A1. A similar blockade was observed with antagonists of the M2 (AF-DX 116, 10 μM, *n* = 8; Fig 5A2), M3 (4-DAMP, 0.5–1 μM, *n* = 4; Fig 5A3) and M4 (tropicamide 1 μM, *n* = 2; Fig 5A4) receptors. At concentrations lower than 1 μM, M1 and M4 antagonists did not suppress the rhythm (*n* = 4). Lower concentrations of M2 antagonist also failed to suppress rhythmic activity (AF-DX 116, 0.5 μM, *n* = 2 and 1 μM, *n* = 2). The effects of selective agonists for M1 and M2 receptors were also tested. M1 agonists (xanomeline and McN-A343) bath-applied to the whole thoracolumbar spinal cord did not elicit any activity at concentrations ranging from 10 to 100 μM (*n* = 2; Fig 5B1, 2). Bath-application of the M2 receptor agonist arecaidine only partly reproduced the oxotremorine effects and induced a weak rhythmic activity, even at high concentration (*n* = 2, Fig 5C1, 2). These data suggest that oxotremorine triggers motor activities in the spinal cord through the activation of the M1, M2, M3, and M4 receptors (see Discussion).

### Slow and fast rhythmic bursting in MNs and IML SPNs

**Fig 6:**
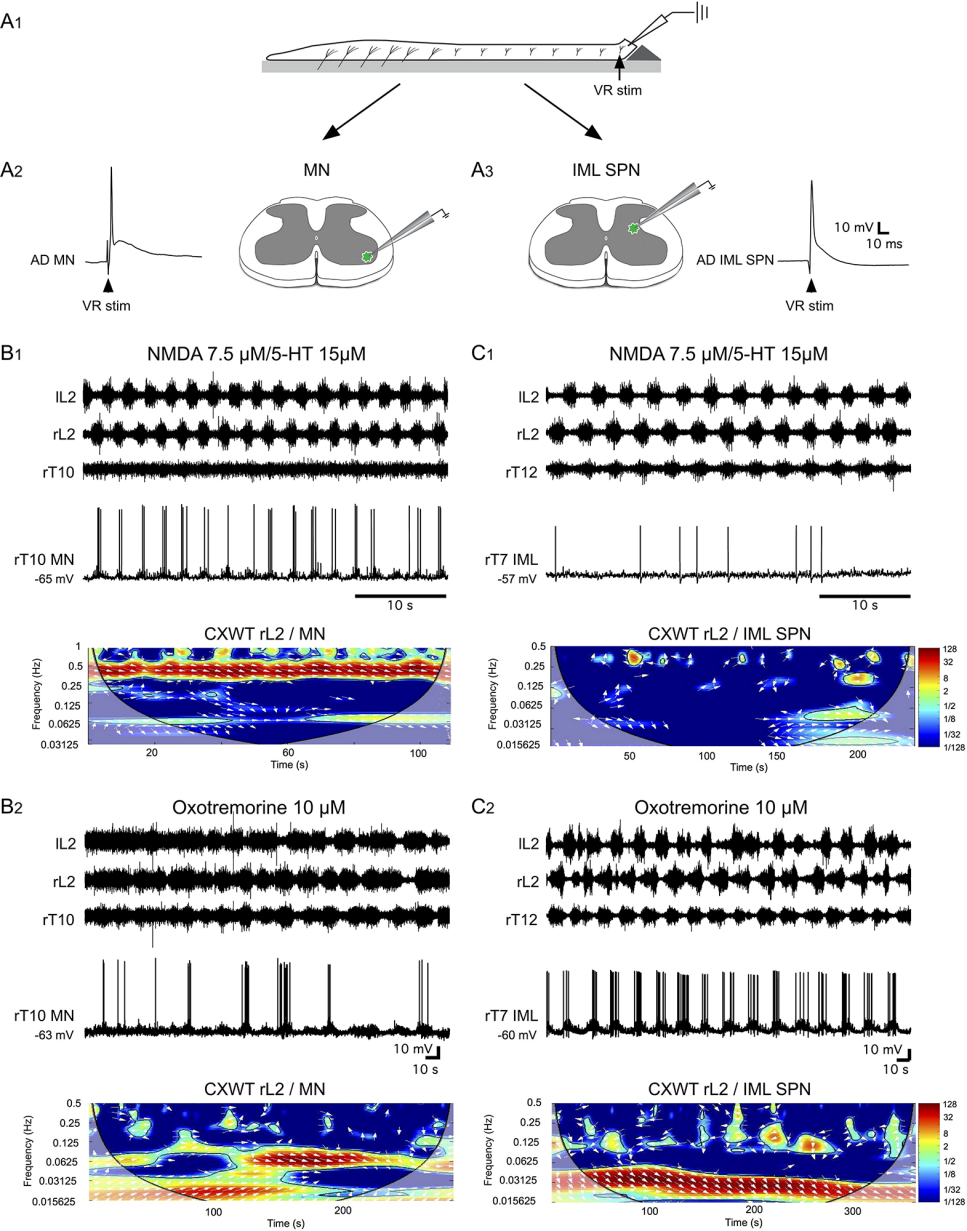
The oxotremorine-induced rhythm drives bursting in both axial MNs and IML SPNs. (A) Schematics of the experimental setup to record MNs and IML SPNs. The cut end of the thoracic spinal cord was upturned on a Sylgard wedge (A1; see Methods) to record from single thoracic MNs (A2) or IML SPNs (A3) identified by their different locations and antidromic action potential firing (AD MN or AD IML SPN) in response to ventral root stimulation of the same segment. (B) Left and right lumbar 2 (lL2, rL2) and right thoracic (rT10) ventral root recordings along with an intracellularly recorded rT10 MN in the presence of NMDA/5-HT (B1) or oxotremorine (B2) on the whole thoracolumbar spinal cord. The mixed cross-coherence maps (CXWT) in lower B1 and B2 show the correlation between the recorded MN's membrane potential oscillations and rL2 activity under NMDA/5-HT (B1) or oxotremorine (B2). (C) Same display arrangement as in B for an intracellularly recorded rT7 IML SPN (rT7 IML). Note that the IML SPN was not driven by the NMDA/5-HT-induced rhythm (C1) but was strongly activated by the oxotremorine-induced rhythm (C2). AD, antidromic; CXWT, mixed cross-coherence map; IML SPN, intermediolateral sympathetic preganglionic neuron; l, left; L, lumbar; MN, motoneuron; NMDA, N-methyl-D-aspartate; T, thoracic; VR stim, ventral root stimulation; 5-HT, serotonin.

Ventral roots not only carry axons of somatic MNs all along the spinal cord but also axons of SPNs from thoracic 1 to lumbar 3 segments in rats. Consequently, extracellular recordings from these latter ventral roots do not permit somatic and sympathetic axonal activity to be distinguished. To assess whether axial MNs and/or SPNs are activated during pharmacological activation of the thoracolumbar spinal cord with NMDA/5-HT or oxotremorine, intracellular recordings from these two cell types were then performed in whole cord preparations (Fig 6A1), in which the MNs (Fig 6A2) or SPNs located in the IML (intermediolateral sympathetic preganglionic neurons [IML SPNs]; Fig 6A3) were identified by ventral root antidromic stimulation. In the majority of recorded thoracic MNs, the bath-application of NMDA/5-HT elicited locomotor-related activity with action potentials superimposed on phasic depolarizations during locomotor cycles (Fig 6B1) [32]. Wavelet transform analysis was conducted to assess whether the extracellular and intracellular activities recorded from the ventral roots and from MNs, respectively, were linked. The mixed cross-coherence map (bottom panel, Fig 6B1) shows that the MN membrane potential oscillations were significantly correlated to the lumbar L2 activity in a frequency band of 0.4 ± 0.02 Hz. In the presence of oxotremorine on the whole thoracolumbar spinal cord, intracellular recordings revealed phasic depolarizations in MNs that were significantly correlated to the lumbar L2 activity (*n* = 22/30 recorded MNs; Fig 6B2). In this sequence, because of the irregular nature of the oxotremorine-induced activity, the frequency band ranged from 0.06 to 0.03 Hz. In contrast, in almost all the IML SPNs tested, no detectable variations in their membrane potential (*n* = 16/18 recorded IML SPNs) were observed in time with locomotor-like activity induced by the bath-application of NMDA-5-HT (top panel, Fig 6C1). The mixed cross-coherence map (bottom panel, Fig 6C1) confirmed that the IML SPN activity was not correlated to NMDA/5-HT-induced fictive locomotion. In contrast, in the presence of oxotremorine, IML SPNs exhibited strong rhythmic burst activity associated with large membrane potential oscillations in correlation with the slow bursting activity recorded from both thoracic and lumbar segments (*n* = 21/25 recorded IML SPNs, Fig 6C2). Wavelet analysis confirmed that in the presence of oxotremorine, IML SPN activity was significantly correlated to the extracellularly recorded neuronal activity in the frequency band of 0.03 ± 0.01 Hz (bottom panel, Fig 6C2).

In conclusion, almost all the MNs and IML SPN neurons tested in these different experimental conditions exhibited similar rhythmic behavior: MNs (73%) were rhythmically activated by both NMDA/5-HT and oxotremorine pharmacological stimulation while IML SPNs (84%) were rhythmically activated solely in the presence of oxotremorine. We call the synaptic command shared by both axial MNs and IML SPNs, in the presence of oxotremorine, the somato-sympathetic drive (SSD).

**Fig 7:**
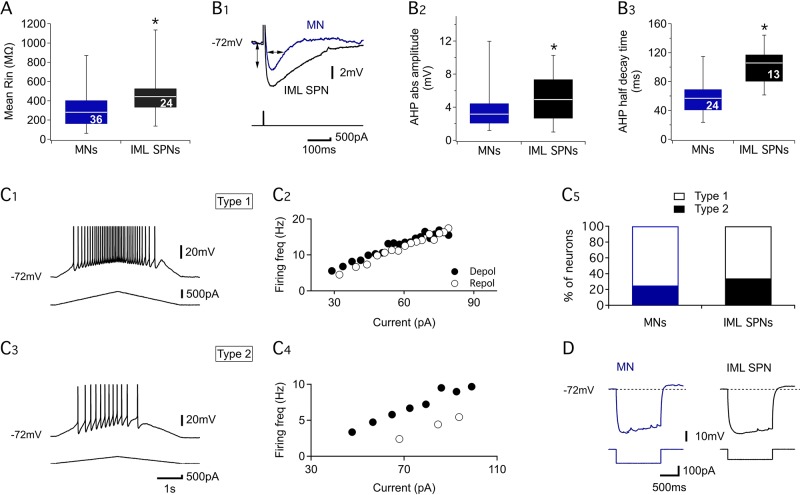
Electrophysiological properties of thoracic MNs and IML SPNs. (A) Box plots of the mean resting membrane potential (Rin) of MNs (blue box) and IML SPNs (black box). (B) Representative traces of the AHP recorded from a MN (blue trace) and an IML SPN (black trace) in response to brief depolarizing current injection (bottom panel) (B1). Box plots of the mean absolute AHP amplitudes (B2) and AHP half decay times (B3) in MNs (blue boxes) and IML SPNs (black boxes). *p < 0.05; numbers in boxes indicate numbers of neurons tested. (C) Membrane potential responses (C1, C3) and firing rate measurements (C2, C4) for two neurons exhibiting linear (type 1, C1, 2) or adaptive discharge (type 2, C3, 4) in response to triangular ramp depolarization (bottom traces in C1, C2). Numbers of MNs (blue bar) and IML SPNs (black bar) expressing type 1 or type 2 firing behavior during triangular current ramps (C5). (D) Representative response of a MN (blue trace) and an IML SPN (black trace) to negative current injections (bottom panel). Note the expression in both neuron subtypes of a hyperpolarization-evoked sag potential and a small post-hyperpolarization rebound. AHP, spike after-hyperpolarization; Depol, depolarization phase; IML SPN, intermediolateral sympathetic preganglionic neuron; MN, motoneuron; Repol, repolarization phase; Rin, resting membrane potential.

Because axial MNs and IML SPNs responded differently to the pharmacological conditions tested, we investigated whether the two subtypes are endowed with different electrophysiological properties that could account for their differing responses (Fig 7). In the absence of pharmacological stimulation, resting membrane potential (RMP) and action potential threshold (AP Th) values were similar between axial MNs (RMP: −61.1 ± 1.2 mV, AP Th: −51 ± 0.7 mV, *n* = 36) and IML SPNs (RMP: −60.4 ± 1.2 mV, AP Th: −49.3 ± 0.9 mV, *n* = 24). However, IML SPNs had a significantly higher membrane input resistance (Fig 7A) and a significantly larger and longer after-spike hyperpolarization (AHP) compared to axial MNs (Fig 7B1–3). Using triangular current pulses and the functional categorization established by Button and collaborators [38], we found that the frequency-current relationships of both axial MNs and IML SPNs could be classified as type 1 (linear, Fig 7C1, 2) or type 2 (adapting, Fig 7C3, 4), with the proportions of these two firing patterns not being significantly different between axial MNs and IML SPNs (Fig 7C5). Finally, we found that both axial MNs and IML SPNs exhibited a sag potential and a post-hyperpolarization rebound in response to hyperpolarizing current steps (Fig 7D). Altogether, these results thus indicate that axial MNs and IML SPNs exhibit basic membrane properties that are relatively similar.

**Fig 8:**
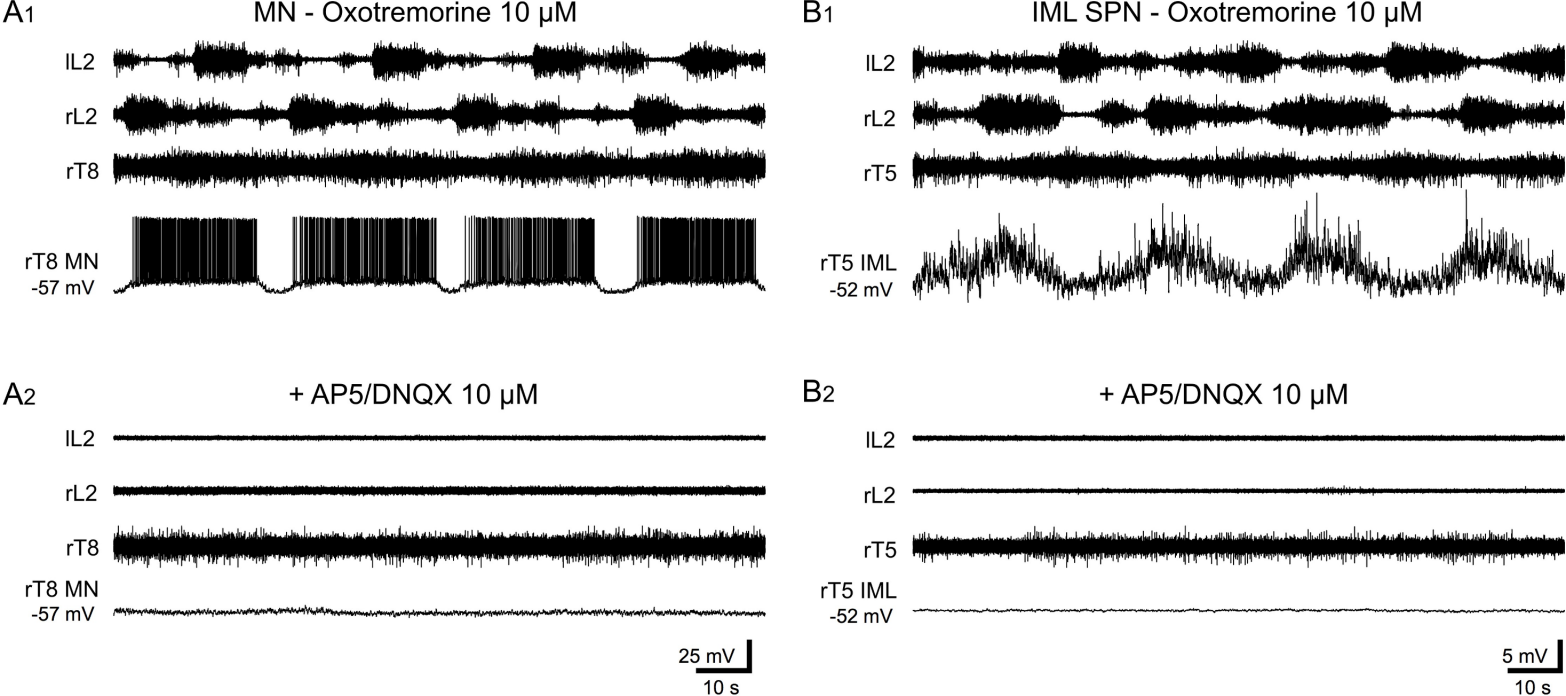
Glutamatergic interneurons are involved in the somato-sympathetic synaptic drive induced by oxotremorine. (A) Recordings from left lumbar 2 (lL2), right L2 (rL2), and right thoracic (rT8) ventral roots and an rT8 MN in the presence of oxotremorine alone (A1) or in combination with the NMDA and non-NMDA receptor antagonists, DNQX and AP5, respectively (A2). (B) A different preparation with lumbar and thoracic (lL2, rL2, rT5) ventral root recordings together with an intracellularly recorded rT5 IML SPN under oxotremorine alone (B1), then oxotremorine plus the glutamatergic receptor antagonists (B2). AP5, 2-amino-5-phosphonovalerate; DNQX, 6, 7-dinitroquinoxaline-2,3-dione; IML SPN, intermediolateral sympathetic preganglionic neuron; l, left; L, lumbar; MN, motoneuron; NMDA, N-methyl-D-aspartate; r, right; T, thoracic.

In a next series of experiments, we investigated the nature of the SSD received by thoracic neurons during the oxotremorine-induced rhythm. In the presence of NMDA and non-NMDA receptor antagonists (10 μM 2-amino-5-phosphonovalerate [AP5] and 10 μM 6, 7-dinitroquinoxaline-2,3-dione [DNQX], respectively), we observed the disappearance of oxotremorine-induced rhythmic bursting in both recorded ventral roots (Fig 8A and 8B) and intracellularly recorded axial MNs (Fig 8A) and IML SPNs (Fig 8B). This suppressive effect, which was consistently observed in the 10 preparations tested, suggested that the spinal network activated by oxotremorine involves glutamatergic neuronal relays that are upstream from MNs and IML SPNs.

**Fig 9:**
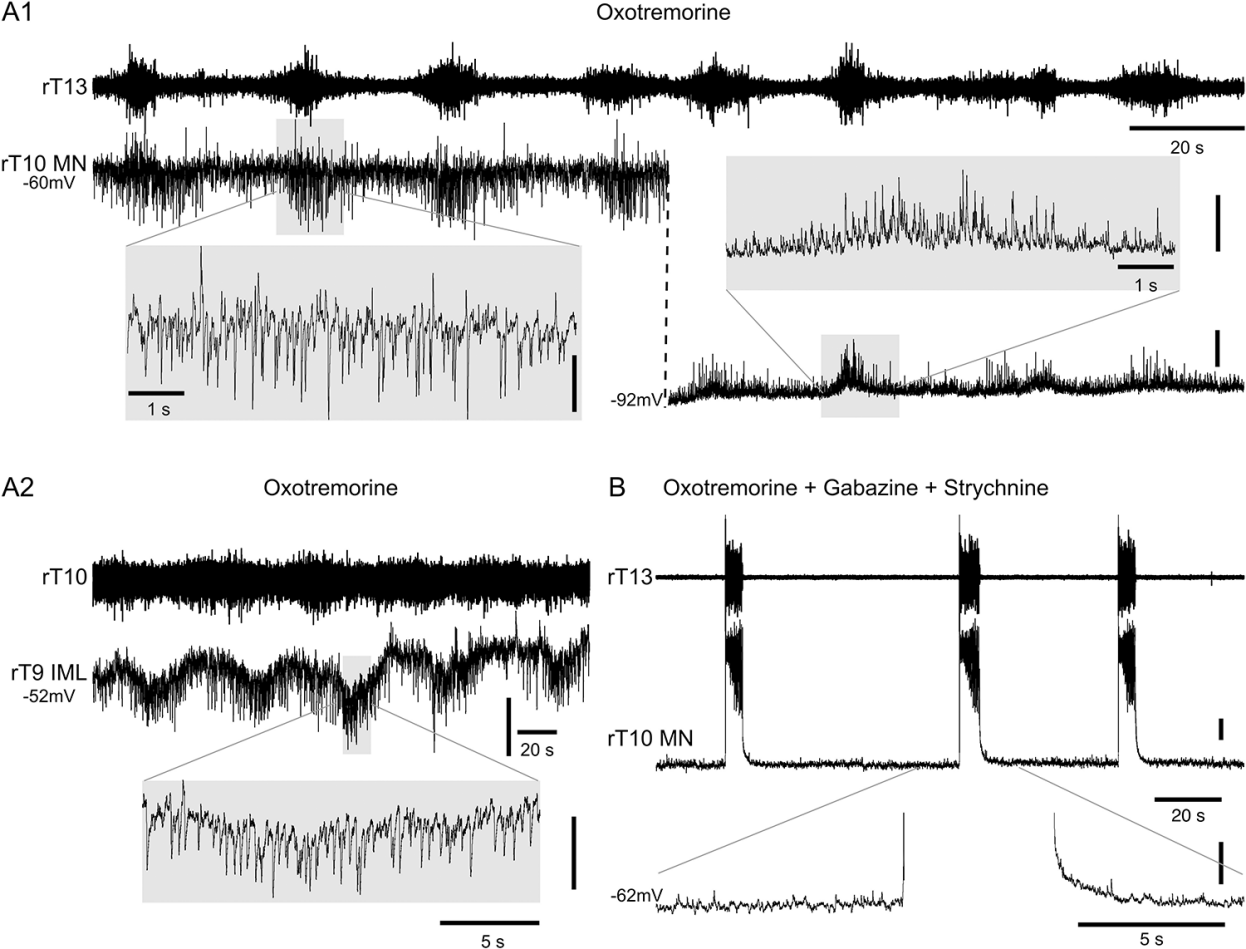
Inhibitory component of the somato-sympathetic drive induced by oxotremorine. (A) Recordings from a right thoracic (rT13) ventral root and a rT10 MN at two different membrane potentials during the bath-application of 10 μM oxotremorine (A1). Note the reversed inhibitory synaptic drive at −92 mV compared to −60 mV. Recordings from an rT10 ventral root and a rT9 IML under oxotremorine (A2). (B) Same MN as in A1 in the presence of oxotremorine and the GABAergic and glycinergic receptor antagonists, gabazine and strychnine (1 μM each), respectively. The gray panels in each case present an enlarged section of the intracellular recording to highlight the inhibitory postsynaptic potentials received by the MN and IML in the oxotremorine condition and their disappearance during the disinhibited rhythm with large spike bursts induced by oxotremorine in the presence of the strychnine/gabazine cocktail. Vertical calibration bars: 5 mV. IML, intermediolateral neuron; l, left; MN, motoneuron; r, right; T, thoracic.

We then assessed whether glycinergic or GABAergic synaptic inputs also participate in the SSD drive, because bursts of inhibitory postsynaptic potentials could be clearly observed (and even reversed) in axial MNs (Fig 9A1) and IML SPNs (Fig 9A2) during the oxotremorine-induced rhythm. However, the glycinergic and/or GABAergic nature of this synaptic drive was difficult to determine, because the addition of strychnine (1 μM) and/or gabazine (1 μM) (antagonists of the glycinergic and GABAergic receptors, respectively) to the oxotremorine-containing saline completely altered the pattern of the elicited rhythm (*n* = 4, Fig 9B) and led to the expression of disinhibited, large amplitude bursting [39]. Nonetheless, together our data suggest that the SSD received by both axial MNs and IML SPNs following mAchR activation involves glutamatergic excitatory neurons and should also implicate inhibitory interneurons.

### Functional coupling between somatic and sympathetic activities

**Fig 10:**
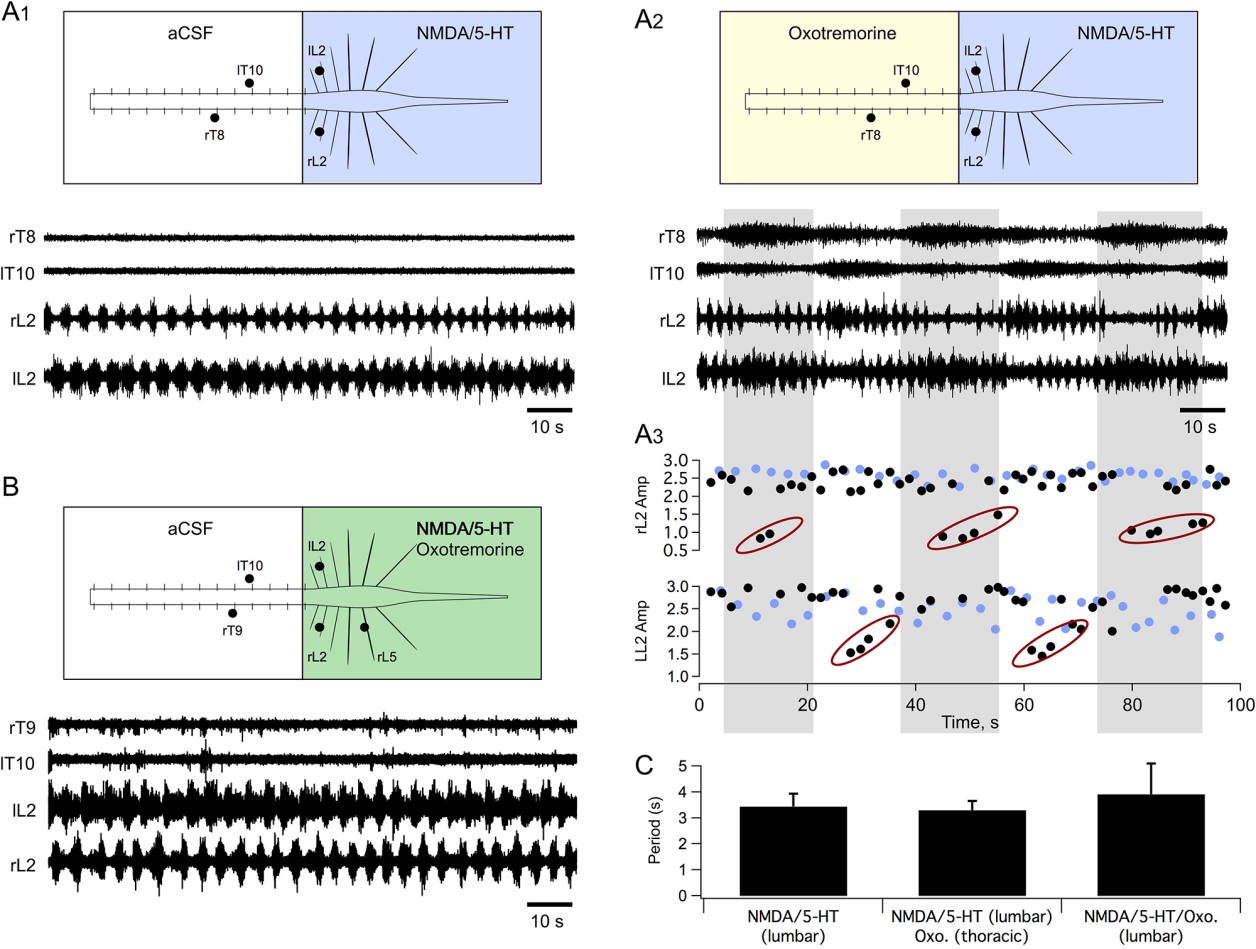
Different influences of oxotremorine-induced rhythmicity in thoracic segments and on lumbar fictive locomotion. (A) In a Vaseline partitioned spinal cord (at the T13 level), bath-application of NMDA/5-HT to the lumbar compartment elicited locomotor-like activity recorded from lumbar ventral roots (A1). Ten micromolar oxotremorine then added to the thoracic compartment induced slow alternating rhythmic bursting in right and left thoracic ventral roots and a strong modulation of the lumbar locomotor-like activity (A2). Cycle-by-cycle plots of burst amplitude in right and left L2 ventral roots (A3). Note that whenever a large burst occurred at the thoracic level, the amplitude of ongoing faster locomotor-like bursts in the ipsilateral lumbar ventral root was strongly decreased (red ovals). (B) The simultaneous bath-application of NMDA/5-HT and oxotremorine to the lumbar compartment failed to trigger slow rhythmic motor bursting in thoracic segments. A and B are from different experiments. (C) Bar plots of the cycle period of fictive locomotion in the different pharmacological conditions tested. aCSF, artificial cerebrospinal fluid; Amp, amplitude; l, left; L, lumbar; Oxo., oxotremorine; NMDA, N-methyl-D-aspartate; r, right; T, thoracic; 5-HT, serotonin.

The above data show that mAchR activation with oxotremorine constitutes a means to unmask the rhythmogenic capabilities of thoracic segments and to recruit both axial MN and IML SPN activity. The question then arises as to the functional consequences of this activation of thoracic networks during the expression of fictive locomotion in the lumbar part of the spinal cord. Fig 10A1 shows representative recordings from a compartmentalized preparation of typical locomotor-like activity induced by bath-applied NMDA/5-HT to the lumbar area only. The subsequent addition of oxotremorine specifically to the thoracic region (Fig 10A2) triggered independent slow bursting activity in the thoracic ventral roots. This slower thoracic burst rhythm was also observed in lumbar ventral roots, where it could be seen to regularly disrupt the ongoing faster locomotor rhythm (Fig 10A2) (*n* = 4/5 tested preparations). The gray columns in Fig 10A2 overlay the motor bursts recorded from the right T8 (rT8) ventral root, which alternated with left T10 bursts. As can be seen in the plots of Fig 10A3, which display the cycle-by-cycle amplitudes of L2 locomotor-like bursts before (blue dots; see Fig 10A1) and during the additional slower rhythm triggered by oxotremorine applied to the thoracic cord (black dots; see Fig 10A2), L2 burst amplitudes were strongly decreased throughout the occurrence of each slow burst in the ipsilateral thoracic segments (see red ellipses in Fig 10A3). In contrast, when oxotremorine was applied with NMDA/5-HT to the lumbar compartment only (Fig 10B), the structure and regularity of the fictive locomotor rhythm remained unaltered, although in 9 out of 12 tested preparations, an overall increase in lumbar burst amplitudes consistent with a previously described direct depolarizing effect of oxotremorine on lumbar MNs was observed [37,40]. Moreover, whether oxotremorine was applied either to the thoracic or lumbar cord region, or to both, the mean cycle periods of the ongoing fictive locomotor bursting were not significantly modified (Fig 10C).

**Fig 11:**
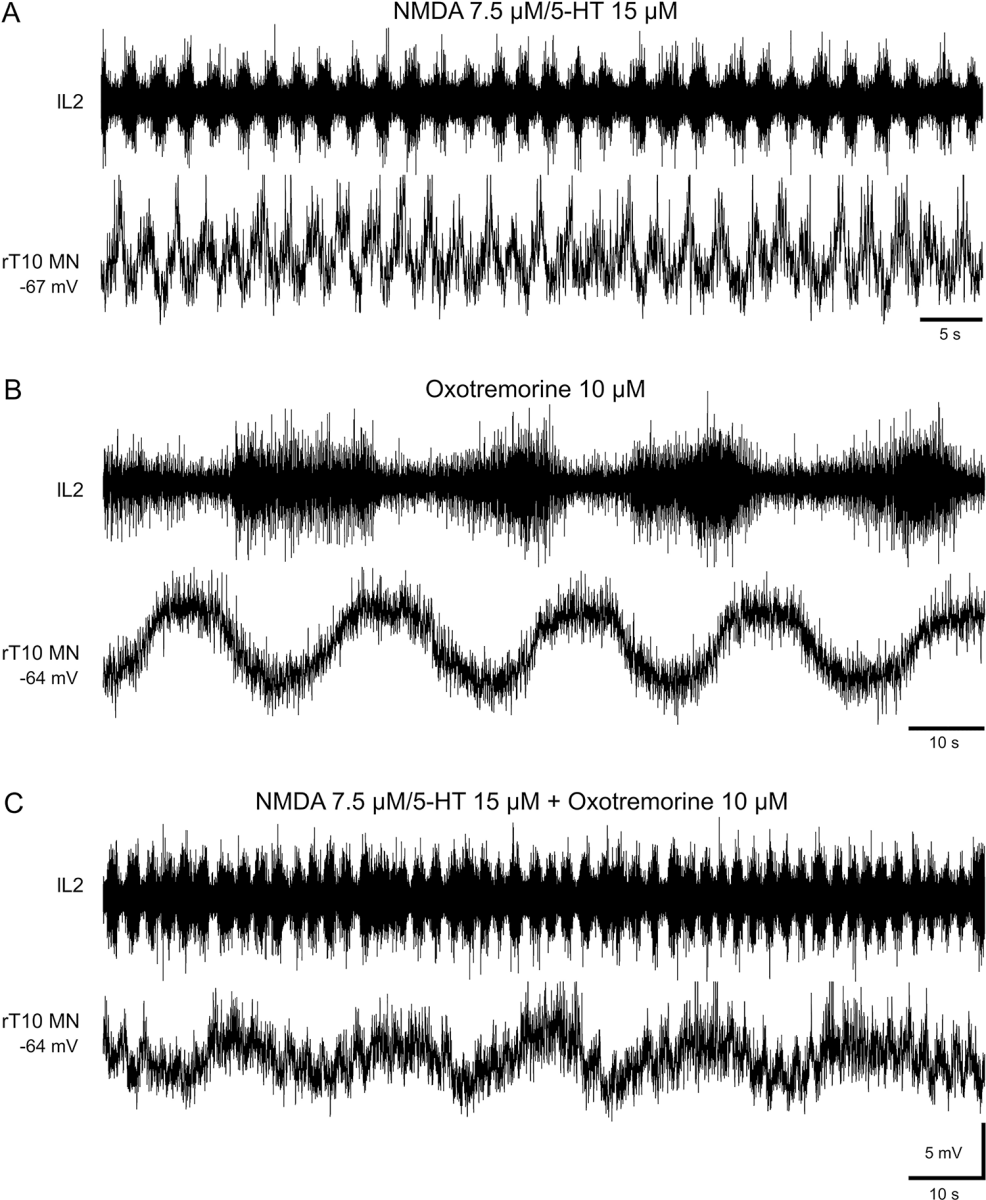
Oxotremorine-induced synaptic merging of slow and fast motor rhythms. Recordings from a left lumbar 2 (lL2) ventral root and a right thoracic (rT10) MN during bath-application to the whole thoracolumbar spinal cord of NMDA/5-HT alone (A), 10 μM oxotremorine alone (B), and NMDA/5-HT plus oxotremorine (C). The conjoint presence of oxotremorine + NMDA/5-HT produced a merging of the slow and fast motor rhythms. l, left; L, lumbar; MN, motoneuron; NMDA, N-methyl-D-aspartate; T, thoracic; 5-HT, serotonin.

Because the axons of both MNs and IML SPNs are conveyed in ventral roots until the L3 segment, we wondered whether the two rhythms expressed in lumbar ventral roots in vitro could somehow be linked to the differential activation of these two neuronal subtypes in vivo. To address this possibility, we made intracellular recordings from thoracic MNs (*n* = 7) under the different neuromodulatory conditions using the same experimental procedures as illustrated in Fig 6. During bath-application of NMDA/5-HT to whole cord, as seen in Fig 6A1, the membrane potential of a recorded thoracic MN underwent rhythmic fluctuations correlated with the locomotor-like bursts monitored extracellularly in the L2 ventral root (Fig 11A; mean cycle period 2.4 ± 0.1 s, *n* = 30 cycles). After washout with normal saline in the same preparation, the bath-application of oxotremorine alone elicited slow rhythmic oscillations in membrane potential correlated with the slow extracellular activity now expressed in the L2 ventral root (Fig 11B; see also Fig 6B2). When both the locomotor-like and slow bursting pattern were simultaneously elicited by the conjoint bath-application of NMA/5-HT and oxotremorine, respectively, a synaptic merging of the two motor rhythms with the superimposition of both fast locomotor-like drive potentials and slow membrane potential oscillations was observed (Fig 11C). Altogether, these data thus indicate that the oxotremorine-induced bursting generated by thoracic networks is able to impose its slower motor-related rhythm on the locomotor activity generated by lumbar networks, resulting in a combined functional coupling of the two motor patterns.

## Discussion

### Motor rhythms generated by muscarinic agonists in the spinal cord

In the present report, we show that the thoracic segments of the newborn rat spinal cord exhibit a preferential sensitivity to mAchR activation compared to the rhythmogenic circuitry in the lumbar segments, leading to the expression of a slow and robust bursting activity. To the best of our knowledge, this is the first demonstration that thoracic networks are endowed with such an independent rhythmogenic capability.

In contrast to the majority of known neuromodulatory systems capable of activating or controlling the motor spinal networks, the cholinergic system does not originate from extrinsic spinal sources but is thought to be completely intrinsic to the spinal cord. While the role of Ach in influencing spinal motor network activities is recognized [21,23,24,26–28,41], its contribution to the generation of the mammalian locomotor rhythm is still debatable [42]. Some of the effects of the cholinergic propriospinal system were also observed in the isolated spinal cord of rodents through the use of AchE inhibitors (such as edrophonium, neostigmine, and physostigmine) that prevent Ach degradation and so enhance the spinal endogenous content of spontaneously released Ach [24,27,42] and increase the potency of bath-applied Ach [21]. In the in vitro spinal cord preparation, AchE inhibitors induce episodes of locomotor-like activity that can be blocked by M2 and M3 muscarinic antagonists [27]. In our experimental conditions with the newborn rat preparation, AchE inhibitors only triggered short bouts of locomotor-like activity. In addition, we observed that the bath-application of oxotremorine systematically and uniquely induced a slow motor rhythm devoid of extensor-flexor alternation, instead of a recognizable locomotor-like activity. Interestingly, this slow motor pattern has been reported in previous studies (see, for example, Fig 1 in Jordan and colleagues, 2014; Fig 1B in Anglister and colleagues, 2008). However, these authors did not describe this slow rhythm and did not test the effects of cholinergic muscarinic agonists. Why do cholinergic muscarinic agonists only partly reproduce the activating effects of AchE inhibitors? Even if muscarinic antagonists abolish the action of AchE inhibitors [27], it cannot be ruled out that either nicotinic receptors are involved or a more localized activation of mAchRs are required to induce locomotor-like activity. Alternately, this may be due to weak potency of available selective mAchR agonists (Ishii and Kurashi, 2006) because the different agonists tested either partly reproduced oxotremorine action or failed to elicit any motor activity.

### Cholinergic activation of MNs and IML SPNs

Ach plays a ubiquitous role in the control of motor and sympathetic outflow. As a neurotransmitter utilized by both the MNs and SPNs, it is engaged in all autonomic ganglia, neuromuscular junctions, and also at many autonomically innervated organs. Furthermore, both MNs and IML SPNs are themselves targeted by cholinergic synapses arising from the cholinergic propriospinal system [20,22,24,29,37,41,43]. The data presented here suggest that the rhythmic activity observed following mAchR activation results from concomitant network and direct effects of oxotremorine on SPNs and MNs. Although the sympathetic nervous system is still maturing at the age studied here [44], Zimmerman and Hochman (2010) previously demonstrated that the membrane properties of IML SPNs are specified as early as P3 and that the overall functional features of SPNs are already mostly in place in the neonate [45]. The present study is the first to provide a direct comparison of the membrane properties of MNs and IML SPNs in similar experimental conditions and reveal no striking differences between these two neuronal subtypes.

### Intraspinal interactions between somatic and sympathetic neuronal activities

MNs as well as SPNs must integrate inputs from both descending and sensory systems that shape the output of the somatic and sympathetic nervous systems. The classical view that has emerged over the past century is that the background activity of SPNs is controlled by supraspinal neuronal networks whose random and diffuse outputs were synchronized by rhythmic inputs arising from cardiovascular and respiratory origins (for review, see [8]). This view was challenged from the late 1970s by the hypothesis that the various rhythmic sympathetic outputs were generated by central neural networks capable of rhythm generation, rather than by pools of interneurons driven by afferent inputs (for review, see [8]). Interestingly, a similar debate emerged at the same time regarding the central generation of motor patterns [46,47].

Double retrograde labeling has identified neurons in the pontomedullary reticular formation, the pedunculopontine tegmental nucleus, and lateral hypothalamus that have been classified as central commanders of both autonomic and motor outflow [48,49]. Some reports have also postulated the existence of a decentralized control system that would reside in the spinal cord [3,5,6,50]. The first evidence for a coupling between somatic and sympathetic outflow in the thoracolumbar spinal cord was provided by Chizh and colleagues [6]. Using an arterially perfused trunk–hindquarter preparation of adult mouse, these authors found that at rest, when only tonic background activity was produced, or during NMDA-induced rhythmic activity, sympathetic and somatic motor output could become synchronized. Subsequently, Goodchild and colleagues (2008) showed that an independent supraspinal coupling exists between multiple sympathetic and motor outflows in the adult rat spinal cord in vivo. These authors proposed that this coupling allows a coordination of activity between the different outflows when hyper-excitation occurs [5]. Here, we propose that an intraspinal rhythmogenic network, whose activation is conditional upon the cholinergic propriospinal system, is likely to be responsible for this synchronizing process. The neuronal substrate of such a network remains elusive (for reviews, see [3,51]). Our data indicate that glutamatergic neurons are part of the local network unmasked by oxotremorine. Potential candidates for the inhibitory component of the SSD is a subset of GABAergic interneurons located around the central canal, which has been previously shown to inhibit SPNs [51,52]. Therefore, further investigation will be needed to assess the different cholinergic, glutamatergic, and inhibitory neurons that compose the rhythmogenic network activated by oxotremorine.

Here, we also found that mAchR activation leads to a superposition of the thoracic and lumbar rhythms induced by the presence of oxotremorine and NMDA/5-HT, respectively. Comparable reconfigurations of motor outputs have been observed in both invertebrate [53] and vertebrate [54] central pattern-generating systems that have been proposed as the basis for the functional flexibility of motor system output.

### Functional importance of intraspinal coupling between somatic and sympathetic activities

Does the slow oxotremorine-induced rhythm have a specific physiological relevance? In physiological conditions, SPNs are rhythmically active in a frequency band ranging from 0.1 to 10 Hz [55]. The slow sympathetic neuronal activities have been related to cardiovascular control [7] and rhythmic changes in arterial pressure (corresponding to the ‘‘10-s rhythm” in humans). In the context of the present study, these so-called “Mayer waves” are of particular interest (for review, see [56]). From their first description, these activity oscillations were referred to as vasomotor waves and were proposed to provide an indirect measure of efferent sympathetic nervous activity. In several species, including humans, Mayer waves are modulated in situations that result in sympathetic activation and parallel the mean level of sympathetic nervous activity [57]. The relationship between the Mayer waves and locomotor (or respiratory) rhythms has been recently explored in decerebrate cats under neuromuscular blockade [58]. These authors reported that the occurrence of Mayer waves was frequently related to the initiation of episodes of fictive locomotion and to variations in the extracellular locomotor burst amplitude. Furthermore, the occurrence of Mayer waves also matched episodes of entrainment between the respiratory and the locomotor rhythms. In the present study, we could not assess whether the intraspinal network revealed by oxotremorine is also involved in respiratory-related discharge concomitant with hind limb muscle activity (as shown by Wienecke and colleagues, 2015). This would require a new experimental paradigm because the isolated spinal cord does not allow us to monitor the respiratory activity that is generated in the brain stem [59]. The frequency range of the oxotremorine-induced rhythm found in the present study is compatible with the frequency of the Mayer waves because, in our experimental conditions, the cycle period of the oxotremorine-induced activity ranged from 13 to 30 s (average period, 21.7 s; Fig 1D1). In our in vitro conditions, however, it has been shown that the locomotor period decreases by more than 50% when the temperature is increased from 25°C (the temperature at which recordings are made) to 35°C [60]. On this basis, therefore, at such higher temperatures corresponding to those in the intact animal, the cycle period of the oxotremorine-induced rhythm would be expected to be less than 10 s.

An important wider implication of the present findings relates to overcoming one of the major problems for people suffering from spinal cord injury (SCI): severe cardiovascular disturbances that contribute to 40% of deaths [61]. The existence of an intraspinal coupling mechanism and the possibility to activate coordinated somatic and sympathetic activities through pharmacological means thus raises the prospect for mitigating vascular dysfunctions after SCI.

## Materials and methods

### Ethics statement

Experiments were conducted in vitro on isolated spinal cords from newborn Sprague Dawley rats of either sex, aged from postnatal day 0 (P0) to P5 (*n* = 108 preparations). All procedures were conducted in accordance with the local ethics committee of the University of Bordeaux and the European Committee Council Directive (approval number 2016012716035720). All efforts were made to minimize animal suffering and reduce the number of animals used.

### In vitro isolated spinal cord preparation

Rat pups were anesthetized with isofluorane until reflexes could no longer be elicited in response to tail or toe pinching. Animals were decapitated, and the skin of the back was removed before preparations were placed dorsal side up in a dissecting chamber. A laminectomy was performed to expose the spinal cord, which was carefully dissected free under a binocular microscope. Dissection and recording procedures were conducted under continuous superperfusion with artificial cerebrospinal fluid (aCSF) equilibrated with 95% O_2_/5% CO_2_, adjusted to pH 7.4 at room temperature (24–26°C) and containing the following (in mM): 130 NaCl, 3 KCl, 2.5 CaCl_2_, 1.3 MgSO_4_, 0.58 NaH_2_PO_4_, 25 NaHCO_3_, and 10 glucose. Spinal cords were sectioned at the T1 level at the beginning of the experiment. In some experiments, the spinal cord was artificially partitioned using Vaseline walls, as previously described [62], to restrict the bath-application of pharmacological agents to specific segmental regions. The watertightness of the barriers was systematically checked at the end of each experiment by observing the movements of methylene blue added to the bathing medium on one side of the Vaseline wall(s).

### Electrophysiological recordings and analysis

Whole cell patch-clamp recordings from neurons were obtained with a Multiclamp 700B amplifier (Molecular Devices). Patch-clamp glass microelectrodes (4–7 MΩ) were filled with the following solution (in mM): 120 K-gluconate, 20 KCl, 0.1 MgCl_2_, 1 EGTA, 10 HEPES, 0.1 CaCl_2_, 0.1 GTP, 0.2 cAMP, 0.1 leupeptin, 77 D-mannitol, 3 Na_2_-ATP, pH 7.3. Intracellular recordings from MNs and SPNs were made according to a protocol developed in a previous study [32]. To access thoracic neurons, a transverse section of the spinal cord was made at a chosen thoracic level using fine scissors (MC-26B, Moria). To maintain the sectioned surface of the cord face-upwards and facilitate microelectrode positioning, the cut end of the spinal cord was positioned on a Sylgard wedge (see Fig 6A1). SPNs and MNs were targeted on the basis of their location in the transverse plane (see Fig 6A) and subsequently identified by recording their antidromic action potentials in response to electrical ventral root stimulation of the same segment (see Fig 6A2–6A3).

A liquid junction potential of +12 mV was experimentally determined [63] and records were corrected for this potential. Series resistance was monitored throughout the experiments and was not compensated. Data were discarded if series resistance varied more than ±20% of the initial value.

Motor activities were recorded extracellularly from various spinal cord ventral roots using glass suction electrodes. Recorded activity was amplified using custom-made amplifiers. The recordings were digitized using an analog-to-digital interface (Heka Elektronik, Germany) driven by Axograph software (Axograph, Australia). Raw signals were high-pass filtered (50 Hz), rectified, and integrated before analysis. Locomotor burst parameters were computed using custom-made routines written in Matlab (Mathworks, France). Mean cycle periods were computed using an L2 ventral root as the reference because it invariably exhibited the best signal-to-noise ratio. Wavelet transform analyses [64] were performed using a Matlab wavelet coherence package provided by Aslak Grinsted (http://noc.ac.uk/using-science/crosswavelet-wavelet-coherence). Of particular interest was the extraction of the common power, correlation, and phase relationship between two simultaneously acquired signals of the cross wavelet transform and wavelet coherence [65,66]. A detailed explanation of the wavelet-based methodology used in the present work has been previously reported [32].

### Pharmacology

Episodes of locomotor-like activity were elicited by exogenous bath-application of a mixture of 7.5 μM NMDA and 15 μM 5-HT [67]. All drugs were obtained from ABCAM. AF-DX 116 (M2 antagonist), 4-DAMP (M3 antagonist), and tropicamide (M4 antagonist) were diluted in DMSO at concentration less than 0.1% [68]. All other drugs were prepared as stock solutions in aCSF.

Drugs were bath-applied using a peristaltic pump (flow rate 4 mL/min; recording chamber volume 4 mL). The effects of the drugs were monitored from 5–10 min after reaching the recording dish (i.e., the time estimated for a total replacement of the bathing saline and diffusion into the tissue).

### Statistics

Statistical analyses of raw data were conducted using GraphPad Prism software. Because of some relatively small samples, nonparametric tests were used for all analyses performed. In the text, all data are expressed as means ± SEM. In the figures, the box plots display the distribution of data based on the minimum, first quartile, median, third quartile, and maximum. Asterisks in the figures indicate statistical significance (*p* < 0.05). Wilcoxon or Mann–Whitney tests were used when applicable. The coupling between left and right activities was analyzed using circular statistics, using the Oriana Software (Kovach Computing Services).

## Abbreviations

Ach: acetylcholine
AchE: acetylcholinesterase
aCSF: artificial cerebrospinal fluid
AHP: after-spike hyperpolarization
AP Th: action potential threshold
AP5: 2-amino-5-phosphonovalerate
DNQX: 6, 7-dinitroquinoxaline-2,3-dione
IML SPN: intermediolateral sympathetic preganglionic neurons
mAchR: muscarinic cholinergic receptor
MN: motoneuron
NMDA: N-methyl-D-aspartate
P: postnatal day
RMP: resting membrane potential
SCI: spinal cord injury
SPN: sympathetic preganglionic neuron
SSD: somato-sympathetic drive
5-HT: serotonin
